# An Experimental Test of Competition among Mice, Chipmunks, and Squirrels in Deciduous Forest Fragments

**DOI:** 10.1371/journal.pone.0066798

**Published:** 2013-06-18

**Authors:** Jesse L. Brunner, Shannon Duerr, Felicia Keesing, Mary Killilea, Holly Vuong, Richard S. Ostfeld

**Affiliations:** 1 School of Biological Sciences, Washington State University, Pullman, Washington, United States of America; 2 Cary Institute of Ecosystem Studies, Millbrook, New York, United States of America; 3 Department of Biology, Bard College, Annandale-on-Hudson, New York, United States of America; 4 Department of Biology, New York University, New York, New York, United States of America; 5 Department of Ecology and Evolution, Rutgers University, New Brunswick, New Jersey, United States of America; Estacion Experimental de Zonas Áridas (CSIC), Spain

## Abstract

Mixed hardwood forests of the northeast United States support a guild of granivorous/omnivorous rodents including gray squirrels (*Sciurus carolinensis*), eastern chipmunks (*Tamias striatus*), and white-footed mice (*Peromyscus leucopus*). These species coincide geographically, co-occur locally, and consume similar food resources. Despite their idiosyncratic responses to landscape and patch variables, patch occupancy models suggest that competition may influence their respective distributions and abundances, and accordingly their influence on the rest of the forest community. Experimental studies, however, are wanting. We present the result of a large-scale experiment in which we removed white-footed mice or gray squirrels from small, isolated forest fragments in Dutchess County, New York, and added these mammals to other fragments in order to alter the abundance of these two species. We then used mark–recapture analyses to quantify the population-level and individual-level effects on resident mice, squirrels, and chipmunks. Overall, we found little evidence of competition. There were essentially no within-season numerical responses to changes in the abundance of putative competitors. Moreover, while individual-level responses (apparent survival and capture probability) did vary with competitor densities in some models, these effects were often better explained by site-specific parameters and were restricted to few of the 19 sites we studied. With only weak or nonexistent competition among these three common rodent species, we expect their patterns of habitat occupancy and population dynamics to be largely independent of one another.

## Introduction

Rodents play important ecological roles in ecosystems ranging from tropical forests to arctic tundra [1]–[5]. By consuming vegetation, seeds, and both invertebrate and vertebrate prey, rodents can profoundly change the structure of terrestrial communities. For example, granivory by kangaroo rats (*Dipodomys* spp.) in the American Southwest determines whether plant communities are dominated by grasses or shrubs, with critical consequences for primary production, the water cycle, and animal community dynamics [6]–[8]. Rodents in tropical and temperate forests can control tree recruitment patterns by their actions as seed predators or seed dispersers [9]–[11]. Voles (*Microtus* spp.) can affect the species composition of herbaceous communities and the rate of tree invasion in grasslands [12], [13]. In temperate deciduous forests white-footed mice (*Peromyscus leucopus*) and eastern chipmunks (*Tamias striatus*) can influence population dynamics of ground-nesting songbirds and possibly of raptors [14], [15]. Rodents can also affect the abundance of multi-host parasites and pathogens [16].

In all of the above examples, the effects of rodents on their communities depend to a large degree on rodent population density. Low rodent density provides seeds, seedlings, adult plants, insects, birds, and other prey an escape from predation; rates of herbivory and predation increase with increasing rodent density. Consequently, the factors governing rodent abundance have generated much interest. Both predators (top-down forces) and resources (bottom-up forces) are known to influence rodent abundance [17], [18]. With the exception of desert rodent communities, however, only modest attention has been devoted to understanding the direct and indirect effects of competitive interactions with other rodents.

In desert rodent communities, populations of many species tend to fluctuate synchronously owing to pulses of primary production and seed availability during rainy years, and reduced seed production during droughts [19]. Synchronous population dynamics would seem to suggest weak or no competition, because competition (all else equal) should lead to negative correlations between abundances of putative competitors. Indeed, in systems dominated by strong bottom-up effects of pulsed resources, competitive interactions can be hard to detect via simple monitoring of population dynamics. Only by integrating experimental species removals with long-term monitoring was it possible to demonstrate that competitive interactions between rodents affect patterns of community assembly, species richness and evenness, and temporal dynamics in arid communities [6]–[8].

Mixed hardwood forests of eastern North America support a guild of granivorous/omnivorous rodents in the families Sciuridae and Cricetidae. Of these rodents, gray squirrels (*Sciurus carolinensis*), eastern chipmunks, and white-footed mice broadly overlap geographically and co-occur locally. All three species fluctuate in response to hard mast such as acorn (*Quercus* spp.) production [20]–[24]. Extensive studies of patterns of presence or absence of these rodent species in fragmented landscapes of the Midwestern United States indicate that each species responds uniquely to landscape and local variables such as forest patch size, isolation, and tree species composition [25]. Despite idiosyncratic responses to landscape and patch variables, patch-based regression models suggest that some species pairs compete and suppress one another's abundances [25], [26].

Although they overlap in diet and other niche dimensions (e.g., shared predators), these species are not functionally redundant. For example, gray squirrels scatter-hoard tree seeds, which favors germination [27], [28], whereas eastern chipmunks and white-footed mice larder-hoard seeds, which typically does not [29]. White-footed mice are voracious predators of pupal gypsy moths (*Lymantria dispar*), a forest pest, but eastern chipmunks are not [30]. White-footed mice support successful feeding by blacklegged ticks (*Ixodes scapularis*) and are efficient reservoirs for Lyme disease spirochetes (*Borrelia burgdorferi*), whereas gray squirrels are poor hosts for ticks and poor reservoirs for *B. burgdorferi*, and eastern chipmunks are intermediate in both respects [16], [31]. Therefore, the consequences of competitive interactions between these rodents are expected to be important in affecting key aspects of the broader communities in which they are embedded.

We designed an experimental study to assess whether gray squirrels, eastern chipmunks, and white-footed mice compete within forested patches of the northeastern United States. We experimentally removed mice or squirrels from small, somewhat isolated forest fragments and added them to others. We estimated the density, apparent survival, and capture probability over the 4.5-month study using mark-recapture models, and measured reproductive effort and mass of each of the three focal species in each forest fragment. We predicted that the density, apparent survival, reproductive effort, and mass of each species would increase as the density of its competitors was reduced, and vice versa.

## Materials and Methods

### Ethics Statement

This research was conducted under the approval of the Cary Institute Institutional Animal Care and Use Committee (protocol 06-01II) in accordance to the guidelines of the American Society of Mammalogists [32].

High resolution digital orthophotos were used to identify 19 forest fragments in Dutchess County, New York, U.S.A. Experimental fragments were small (0.63 ha to 11.9 ha), isolated from other forested areas (≥80 m to nearest forest edge), and at least 1 km apart to maximize independence of rodent populations therein. Sites were grouped into clusters of two or three by proximity, then each site within a cluster was randomly assigned to one of five treatments: mouse removal (n = 4), mouse addition (n = 4), squirrel removal (n = 4), squirrel addition (n = 4), or unmanipulated controls (n = 3) (see Table S1 for site assignments and pairings).

In each forest fragment we set up a grid of Sherman traps (7.6×8.9×30 cm) spaced 15 m apart, with 16 Tomahawk traps (15×15×48 cm) on alternating rows and columns. We tried to fit an 8×8 grid in each site (4×4 for Tomahawks), or at least a rectangular array, but the shape and size of grids varied to accommodate the shape of the fragment. Trapping grids encompassed areas from 0.3–1.24 ha and 21–66 (mean 48.6) Sherman and 6–18 (mean 13.3) Tomahawk traps. In the four largest removal sites (sites 36, 40, 209, and 1009) we included an additional buffer strip of up to 30 m with three to 30 additional traps (Shermans on mouse removals, Tomahawks on squirrel removals, nothing on controls or sites that were too small). These buffer traps were used to help reduce densities of the target species and intercept immigrants before they reached the central grid, but the captures in them were not used in the analyses reported below.

In order to reduce the initial densities of focal species on the removal sites, we conducted a trapping surge only in the removal sites for eight days over two weeks starting May 19, 2009. Any mice (from mouse removal sites) and squirrels (from squirrel removal sites) that were not lactating were removed to a non-experimental site.

From June 2 until late September sites were trapped for two consecutive days ( = one trapping session)—removal sites on Tuesdays and Wednesdays, addition and controls sites on Thursdays and Fridays—every week with a few exceptions, noted in Protocol S1. Sherman traps were baited with crimped oats and Tomahawk traps with whole walnuts, set between 3:30 pm and 5:30 pm, and checked between 8:30 am and 12:00 pm the following morning. Upon initial capture, animals were given a numbered ear tag (squirrels were given two: one in each ear) for unique identification. On the first capture in a trapping session each animal was weighed to the nearest gram, sexed, assessed for reproductive status (scrotal testes for males, pregnant or lactating for females), and mice were aged according to the pelage coloration (juvenile, subadult, adult).

In removal sites, every individual of the appropriate species was removed upon capture (unless it was lactating, in which case it was released at the site of capture) and transferred to its partner addition site (Table S1). The proportion of mice and squirrels that were lactating did not differ among treatments; *P*>0.342. Any ticks (*Ixodes scapularis*) on an animal were removed with forceps before the animal was transferred to a new site (tick data to be presented in a companion study). Animals were transported in the trap in which they were captured, supplemented with apple slices for hydration, and then released at the center of the recipient addition site as soon as trapping at the removal sites was completed.

We analyzed the mark-recapture data in two steps. First, we estimated the abundance (*N*) of mice, squirrels, and chipmunks (the latter not manipulated, but still a species of interest) separately using the closed population robust design [33], [34], which allows for immigration and emigration between trapping sessions, but assumes the population is closed within a session. Sites trapped on the same days of the week were analyzed together. All eight removal sites and control site 2709, which were trapped on Tuesday and Wednesday, were analyzed as a group, which we call the “A” sites. All eight addition sites and the remaining two control sites, which were trapped on Thursday and Friday, were analyzed as a group and called the “B” sites. In this way model parameters for sites with few captures or recapture (e.g., removal sites) could be informed or constrained by data from other sites and thus be estimated with more precision, while allowing parameters such as capture probability (*p*) to vary with quickly-changing conditions, such as weather, which could change dramatically from Tuesday (A sites) to Thursday (B sites). This also allowed us to look for consistent results between these more or less independent sets of trapping sites.

We fit a suite of 12 closed population robust design models to the A and B sites separately for each of the three species. In these models, apparent survival (*S*) and capture probability (*p*) were either constant, varied among sites, or varied with the treatment. Capture probability could also vary by site. Recapture probability (*c*) was equal to *p* plus a constant. While models allowing for temporary emigration (i.e., temporary unavailability for trapping) and immigration (*γ*″ and 1 - *γ′*, respectively) were favored by Akaike's Information Criteria, adjusted for sample size (AICc) [35], we set these parameters to zero to avoid issues with parameter identifiability and to make estimates of abundance more precise. Setting these parameters to zero had the effect of deflating estimates of *p* and *c* relative to the model with temporary emigration, and thus inflating estimates of *N* by about 20% for squirrels and chipmunks, and slightly less for mice, but this bias was consistent among sites and so should not change our results. We report the density of each species (*N/*ha) on each of the 19 sites during each trapping session produced by the model with the lowest AICc value for each species, although the estimates varied little among models.

In order to test whether the density of each species was a function of the densities of its putative competitors, we first calculated the mean densities of each species at each site during the first, middle, and last four weeks of regular trapping (June 6–30, July 14–August 6, and September 9–29, respectively). We then regressed the final densities of a given species (e.g., mice) against its initial density plus the densities of the two other species (here chipmunks and squirrels) in the middle or end of the experiment. Our expectation was that the final density of mice, for instances, would be positively related to their initial density, but negatively related to the densities of chipmunks and squirrels several weeks before. We used quantile regression of the 10th–90th quantiles (by tens) to provide a fuller view of how the densities of putative competitors might influence the focal species [36]. We might, for instance, expect that the maximum densities attainable (i.e., the higher quantiles) would be reduced in the face of higher competitors densities, even if the mean or median effects were very small. Given our relatively small sample size (n = 19 sites), we used bootstrap confidence intervals to evaluate the deviation of parameters from zero. Models with proportional changes in density as a response variable produced equivalent results and are not reported.

Second, we estimated the effects of the abundance of other species—potential competitors—on the apparent survival and capture probability of the focal species (e.g., the effect of squirrels and chipmunks on the survival and capture probability of mice) using the Huggins parameterization of the closed population robust design. In this formulation, the population size (*N*) is factored out of the likelihood, so there are fewer parameters to estimate. We used the same suite of models as above, but in order to examine the effect of potential competitors on the capture probability (*p*), which we interpreted as a measure of activity, and apparent survival (*S*) of each species, we made these parameters a logistic function of the number of competitors on the grid during each trapping period. For instance, the apparent survival of mice might change with the number of chipmunks and squirrels as logit(*S*_mice_) = *β*_0_+*β*_1_×*N*_chipmunks_+*β*_2_×*N*_squirrels_. We use both *N* or ln(*N*+1) since we had no *a priori* reason to expect linear or less-than-linear responses to increasing competitor abundance. The parameters *p* and *c* were fixed at zero for the dates that certain sites were not trapped (see Protocol S1) and *γ*″ and *γ*′ were estimated as separate constants, representing Markovian emigration and immigration.

Lastly, we examined how the mass of each species changed as a function of the density of intra- and inter-specific competitors. Since these three rodent species are short-lived there were very few individuals found both at the beginning and end of the experiment. Instead we used linear mixed models to look for general trends in mass. We restricted our analysis to non-lactating, non-pregnant adults to avoid the confounding effects of mass gain and loss associated with both development and reproduction (N = 2860 observations of 990 mice, 889 observations of 302 chipmunks, and 390 observations of 159 squirrels). We allowed each individual to have a random intercept and slope in our models. We also included the main effect of date (centered on the midpoint of the experiment to improve convergence) to account for any general trends of weight loss or gain. We then added to this base model the main effects of the densities of the three species averaged over the whole experiment. (The results do not change if we use only the densities from the initial or middle four weeks of the experiment.) We used AIC and estimates of the regression coefficients to determine whether models with competitor densities better fit the data than the base models with only conspecific densities.

We used the R [37] package RMark [38] to construct and analyze models in Program MARK [39], the quantreg package [40] for quantile regressions, the lme4 package [41] for linear mixed models, and the ggplot2 package [42] for graphs.

## Results

Throughout the four months of regular trapping we captured a total of 716 individual mice on the A sites (eight removal sites and one control site trapped early in the week). In the four mouse removal sites we removed 351 individual mice (excluding 150 mice removed from the buffer strip around site 40) leaving four lactating females, five small, dependent juveniles, and nine mice caught only on the first day of the last trapping session when we were not removing animals. A total of removed 326 mice were added to one of the four addition sites. On the B sites (ten addition and two control sites trapped late in the week), we captured 885 individual mice including 117 that had been added from the removal sites. Mice were being removed and added throughout the entire four-month period.

We captured 90 squirrels in the control sites and 40 on the squirrel removal sites (excluding 12 captured and removed from the buffer strips around these sites), 38 of which were relocated to the addition sites; one of the remaining squirrels died and the other was a lactating female. On the B sites we captured 94 individual squirrels, 11 of which had been added. Most squirrels were captured and moved between June and mid-July. Lastly, we captured 168 individual chipmunks in the A and 162 in the B sites. (Chipmunks themselves were not manipulated).

**Figure 1:**
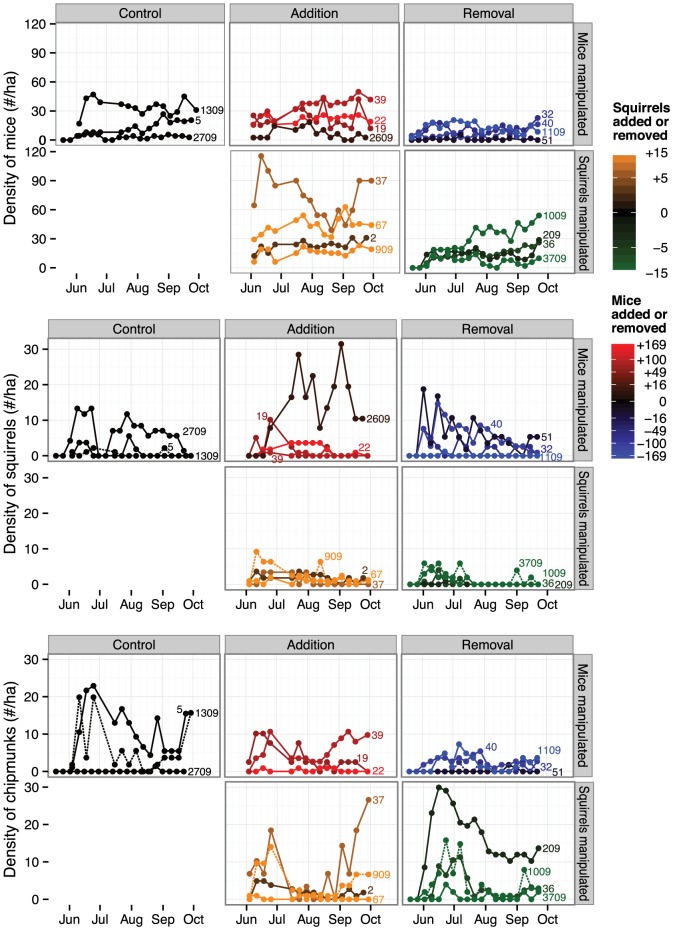
Population dynamics of small mammals according to treatment. Estimated densities of white-footed mice, gray squirrels, and eastern chipmunks in 19 forest fragments in Dutchess County, New York throughout the study. The numbers next to the lines represent the forest fragment identity. Fragments are grouped in panels according to whether the site was in the control, mouse addition, mouse removal, squirrel addition, or squirrel control treatment. Confidence intervals were omitted for clarity. Note that the dashed line type is used only to help clarify overlapping population trajectories.

The estimated densities of each species were comparable to those observed in similar forests in the Northeast (e.g., 9.5 to 28 *P. leucopus*/ha in northern Connecticut, [43]; and 3 to 11 *S. carolinensis*/ha. in Baltimore, [44]) and our own long-term data from the Cary Institute (0.1 to 80 *P. leucopus*/ha., 0 to 33 *T. striatis*/ha., [45], and 0 to 5.77/ha; RSO unpublished data). The densities of each species varied considerably through time on at least some sites (Fig. 1). Adding squirrels to sites did not noticeably increase the density of squirrels on addition sites (Fig. 1). Most added squirrels did not stay or survive on these sites—of the 38 squirrels added, just 11 (29%) were captured on the new site, only seven more than once (three on site 909, two on site 67, and one on the other two addition sites)—but those that remained made up a sizeable fraction of the captures—roughly half of the captures on sites 909 and 67, and four out of five on site 37. There was no difference in the number of captures of addition and resident mice on the addition sites (an average of ∼2.5 captures per squirrel; Poisson generalized linear model z = 0.576, *P* = 0.564).

The addition of 88 mice to site 29 led to a noticeable increase in density (Fig. 1); 37 (42%) of which were captured at least once, 18 (20%) at least twice, and ten (11%) at least three times. Some of these added mice clearly became residents of their new forest fragments—one was recaptured 23 times over a period of 113 days. In the month of September 15% of all captures were of added mice. On other sites the manipulation was less successful (Fig. 1). For instance a total of 169 mice were added to site 22—more than ten mice were added in each of five weeks—yet the population never exceeded 30 on this site. Many of the added mice became residents: 74 (44%) of the added mice were captured at least once on the new site and 41 (24%) at least twice. In September 51% of the captures on site 22 were of added mice. Site 19 had a total of 62 mice added, just six of which were captured more than twice, although these represented 38% of the captures in the last month of trapping. Only seven mice were added to site 2609 and there was no noticeable increase in abundance. Across all mouse addition sites, added mice were captured significantly fewer times than resident mice (an average of 3.21 vs. 4.56 captures per mouse, respectively; negative binomial generalized linear model z = −6.399, *P*<0.0001).

The experimental removals were more successful, especially for squirrels. The early removal of 14 squirrels from site 1009, 15 from site 3709, and seven from site 36 reduced squirrel populations on these removal sites to zero or near-zero levels. Only two squirrels were ever captured from site 209 and they were removed. Our removals of mice seem to have kept mouse populations low as well. We removed 177 mice from site 1109, 101 mice from site 40, and 66 mice from site 32. Roughly half of the mice captured and removed from these sites after the initial surge were in adult pelage although up to 15% on a site were captured as juveniles, suggesting that our removals were being counteracted primarily by immigration, but also by recruitment. Some control sites and sites in which mice were not manipulated (e.g., 2709, 3709, and even the mouse addition site 2609) had densities at or below some of the mouse removal sites (Fig. 1).

**Figure 2:**
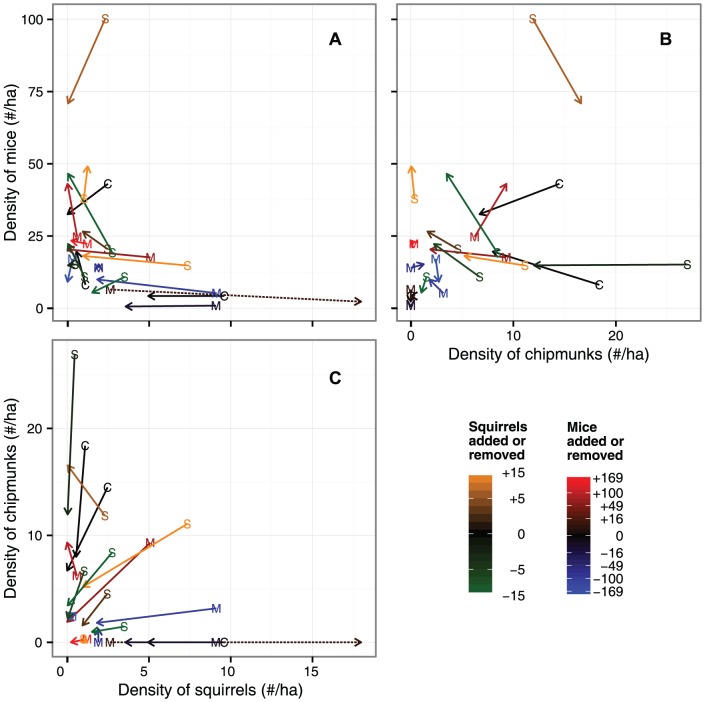
Population trajectories among species pairs. Change in mean densities between mice and squirrels (A), mice and chipmunks (B), and chipmunks and squirrels (C). The letters correspond to the initial mean abundance over the first four weeks of regular trapping and the arrowhead to the mean abundance over the last four weeks in each of the 19 forest fragments in Dutchess County, New York. Letters and line color and type indicate the treatment of each site. Note that the dashed line type is used only to help clarify overlapping population trajectories.

The initial densities of mice and squirrels among sites during the first month of trapping were strongly negatively correlated (Spearman's *ρ* = −0.484, *P* = 0.037; Fig. 2a) while the initial densities of mice and chipmunks were not significantly correlated (*ρ* = 0.419, *P* = 0.075). The magnitude and direction of these correlations persisted to the last month of trapping (*ρ* = −0.644, *P* = 0.004 and *ρ* = 0.417, *P* = 0.077, respectively). In addition, the correlation between chipmunks and squirrels, which was initially small and not significant (*ρ* = −0.265, *P* = 0.272), became strongly negative in this final month (*ρ* = −0.705, *P* = 0.001).

The upper quantiles of the densities of each species at the end of the study were positively related to their initial densities (i.e., sites with more mice initially tended to have more mice by the end of the experiment). This was true for mice and chipmunks across all but the 70th (mice and chipmunks) and 80th (chipmunks) quantiles, and while apparent in the higher quantiles for squirrels was only significantly different from zero at the 60th and 70th quantiles. Final densities of the three species, however, were unrelated to the densities of their putative competitors in the middle or final month of trapping across the whole range of quantiles, with one exception: the 90th quantile of chipmunk density was significantly *positively* related to the densities of mice in middle month of the experiment (*β* = 0.168, *t* = 2.426, *P* = 0.028). In other words, higher mouse densities predicted higher densities attainable by chipmunks in the following months.

The overall lack of numerical responses to our manipulations or competitor densities was underscored by the patterns of reproductive effort, as measured by the proportion of females that were pregnant. A significantly smaller fraction of mice were found pregnant in the mice removal sites (15%) than in the control sites (29%; *t* = −2.698, *P* = 0.017), perhaps because they were removed before they could find a mate or their pregnancy became visible. No other treatments, including the addition of mice or squirrels, significantly altered reproductive effort (all *P*>0.273), nor was mouse reproductive effort associated with chipmunk or squirrel density (all *P*>0.586). Only two pregnant squirrels and seven pregnant chipmunks were observed, so similar comparisons were not possible.

The mass of non-lactating, non-pregnant adult mice (by pellage) increased on average by 2.8 g over the duration of the experiment (*β* = 0.021, *t* = 8.62), and more so at higher mouse densities (*β*_mice_ = 0.035, *t* = 5.49). Adding competitor densities to this base model did not improved the fit (ΔAIC = 1). (Note that the lme4 package does not return P-values because of the controversy surrounding how or whether it is possible to estimate the denominator degrees of freedom in all but the most restricted mixed models. With large sample sizes we can assume that the t-test statistics should converge on the normal, so we employed a cutoff of ∼2 as being significant [46]). Chipmunk mass also tended to increase with time (*β* = 0.037, *t* = 3.38), but the individual patterns of weight gain/loss were also much more variable (standard deviation of random slopes = 0.120). There was very little improvement in model fit with the addition of the main effects of competitor densities (ΔAIC = 0.2). Squirrel mass tended to *decrease* with time (*β* = −0.264, *t* = −2.66), but again this effect was small relative to the random variation in slopes (standard deviation of random slopes = 0.632). Adding competitor densities led to a *worse* model by the AIC criterion (ΔAIC = 4.3).

**Figure 3:**
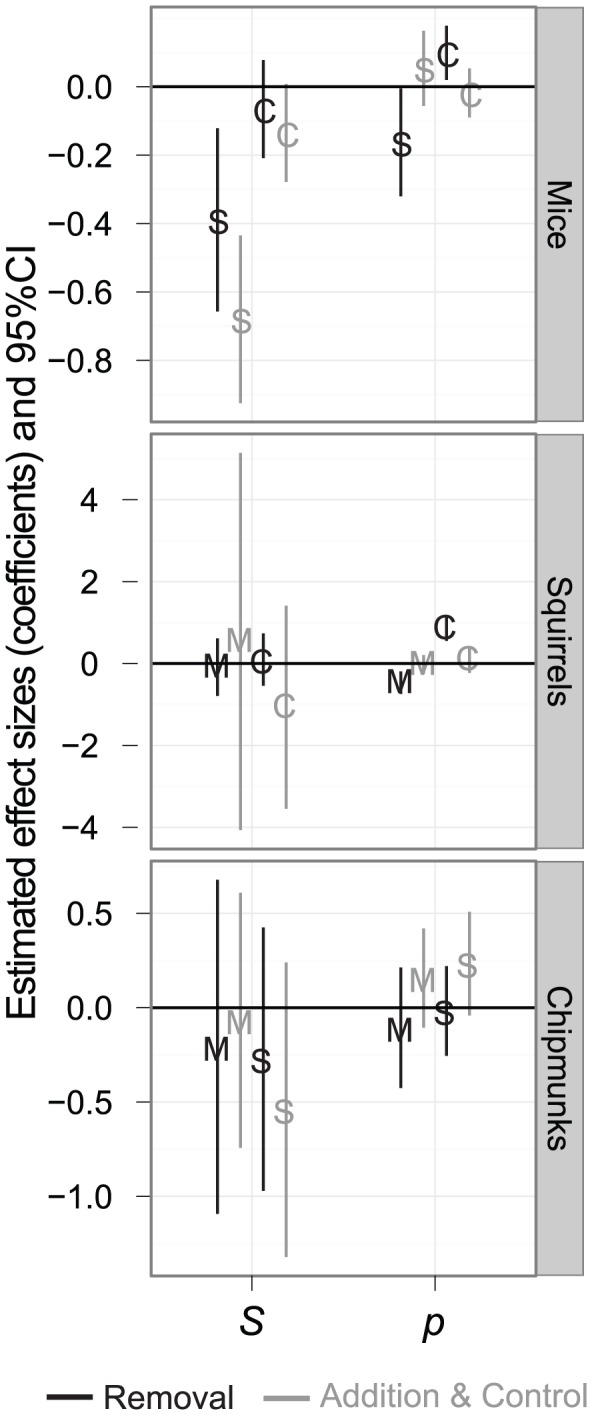
The effects of competitor abundance on capture probability and apparent survival. Estimated effects sizes (model-averaged coefficients from mark-recapture models) of the abundance (natural-log transformed) of putative competitors (M = mice, S = squirrels, and C = chipmunks) on the capture probability (left) and apparent survival (right) of mice (top panel), squirrels (middle panel) and chipmunks (bottom panel). Coefficients from models fit to A sights are black, and those fit to B sites are grey. Vertical lines are 95% confidence intervals. Note that parameters are plotted on the logit scale.

There was little consistent evidence that the abundance of potential competitors reduced the apparent survival (*S*) or altered capture probability (*p*) of the focal species. Among the models fit to the mouse datasets, those in which survival rates were site-specific were strongly favored (>99.9% evidentiary weight; Table S2). Specifically, the best-fit models were *S*(site) +*p*(session+*c*) for the A sites and *S*(site)+*p*(site+*c*) for the B sites. While less than 0.1% of the evidentiary weight fell behind models that included the abundance of squirrels and chipmunks, these were moderate (A sites; ΔAICc = 5.4) to large (B sites; ΔAICc = 36.6) improvements over models with constant survival (Table S2). In these models the apparent weekly survival of mice was predicted to decrease from a high of 0.88 in the A sites when squirrels were least abundant to 0.71 when squirrels were most abundant, and similarly in the B sites from 0.92 to 0.68 (coefficients for these models are shown in Fig. 3). Capture probability of mice also decreased slightly with increasing abundance of squirrels and increased with the abundance of chipmunks, but these effects were only seen in the A sites (Fig. 3). Again, there was negligible evidentiary support for these models. Moreover, when models were fit individually to each of five sites with a substantial variation in squirrel abundances (sites 22, 40, 909, 1009, and 2609), the best model included squirrel abundance in just two of these five datasets, and in only one case (site 22) were the parameter estimates for the effect of squirrels significantly different from zero (*β_ln_*_(squirrels)_ = −0.607, 95% CI = −1.115 to −0.098).

Focusing on squirrels, models in which capture probabilities were a function of the abundance of mice and chipmunks in a site comprised almost 99% of the evidentiary weight in the dataset comprised of A sites (Table S3). The best-supported model, with 79.2% of the weight, was *S*(.) *p*(*ln*(mice)+*ln*(chipmunks)+*c*). (Parameters that are constant are represented by “(.)”.) In these A sites, model-averaged estimates of capture probability increased substantially with the abundance of chipmunks—from 0.11 to 0.43 over the range of chipmunk abundances, holding mouse abundance constant—and decreased substantially with mice (Fig. 3)—from 0.80 to 0.16 across the range of mouse abundances. Accounting for the abundance of both species, capture probability was predicted to vary from a low of 0.07 to a high of 0.74, with a mean of 0.27. In order to test whether these relationships between squirrel capture probability and the abundance of their putative competitors reflects interactions within a site, as opposed to correlations across sites, we chose three sites (40, 51, and 2709) where there were sufficient numbers of squirrel, mouse, and chipmunk captures with which to estimate these effects and fit the models to each site individually. In two of these (sites 40 and 2709) the best-fit model included capture probability as a function of *ln*(mice), but these were not substantially better than models with a constant *p* (ΔAICc = 3.92 and 1.06, respectively) and in only one of these (site 40) was this effect significantly different from zero (*β_ln_*_(mice)_ = −0.535, 95% CI −0.948 to −0.121). Moreover, there was essentially no evidentiary support for models with a similar effect of competitors on squirrel capture probability or apparent survival in the B sites (Table S3; Fig. 3). In the B sites the best-supported model, with 68.7% of the evidentiary weight, was *S*(.) *p*(*site*+*c*).

In both chipmunk datasets, approximately 20% of the evidentiary weight fell behind models in which apparent survival was a function of competitor abundances (Table S4). None of these parameters, however, were significantly different from zero (Fig. 3).

## Discussion

We set out to experimentally test whether three widespread rodents compete with each other in mixed hardwood forests of the Northeast, removing white-footed mice and grey squirrels from some forest fragments and adding them to others in order to alter their local abundances. Despite the continuous removal and addition of several hundred mice and dozens of squirrels for over four months our manipulations, especially the addition treatments, did not alter abundances greatly. Only one of four mouse addition sites and none of the squirrel addition sites showed marked increases in abundance (Fig. 1). Some mice and squirrels were recaptured multiple times in the sites to which they were added and made up a large fraction of the overall captures, suggesting that they became residents, although fewer than we expected from previous studies [43]. Territorial defense by residents might account for our inability to increase the density of mice in these fragments [43], [47]. Density-dependent territoriality is common in white-footed mice (but not in gray squirrels [48], [49]) and can prevent experimental or natural immigrants from becoming established [43], [47]. Indeed, recapture rates were lower for added mice than resident mice (but not for squirrels). Added animals that became residents would seem to have done so by evicting residents with little net change in density, all of which highlights the strength of intraspecific interactions.

We were better able to reduce or maintain low abundances of mice and squirrels on the removal sites, even with continual immigration from outside of our trapping grids. However, some unmanipulated control sites had similarly low densities. We conclude that the resulting rodent abundances in these fragments were less a product of our manipulations than of local conditions (e.g., resource levels) or some unknown feature(s) of the fragments. Whatever the cause, there was substantial variation in the abundances of mice and squirrels among our 19 forest fragments with which to look for patterns consistent with competition.

Overall, we found only weak and inconsistent evidence of competition. For instance, while there was a strong negative correlation between the initial densities of mice and squirrels across fragments (and a negative, but non-significant correlation between chipmunk and squirrel densities), this could be the outcome of competition past or, alternatively, a situation in which sites that are better for mice are worse for squirrels, and vice versa. More direct evidence of competition would be strong increases in the density of a species when its competitors were reduced. For instance, two squirrel removal sites showed strong increases in mouse density from the start to the end of the experiment (Fig. 2a). However, the other two squirrel removal sites saw no increase in mouse density, nor did several other sites that saw large (natural) reductions in squirrel populations, which suggests that mouse populations were independent from the dynamics of the squirrels. In general, the densities of squirrels and chipmunks declined over the experiment, regardless of treatment or density of their putative competitors. Mouse populations, on the other hand, tended to increase or stay relatively constant. Just two sites had strong declines in mouse density, but these were not associated with increases in either of the other two species.

A more formal test of demographic responses to competitors across all of the fragments using quantile regression yielded similar results. Quantile regression accounts for the fact that many factors can influence the density of a species in addition to competitor densities [36], but that high densities might put an upper limit on the density a given species might attain in a fragment. We found that while the initial density of a given species was important in predicting its final density (i.e., dense populations tended to remain dense, and sparse populations sparse), particularly at the upper quantiles, the densities of the competing species were not similarly predictive. The one exception was the final chipmunk density, which increased with mouse densities, opposite of what we would expect from competition.

It is possible that the numerical responses to competition did not have time to materialize during our study, but while we cannot exclude this possibility, it does not seem likely. Mice, for instance, could have had several litters during the removal/addition period and even mice born two months into the study would have been reproductively mature by the end. We found no differences in reproductive effort (the proportion of female mice that were pregnant or lactating) with competitor densities. Moreover, immigration was clearly occurring and could have led to noticeable increases in densities. Indeed, immigration accounted for most of the numerical responses to removals and food supplementation in previous experiments with other rodent species [50], [51].

In addition to a lack of numeric or reproductive responses to competitors, the effects of competitor densities on apparent survival and capture probability were also weak and inconsistent. The apparent survival of mice in one model, for instance, declined with increasing abundances of squirrels—up to an estimated 25% reduction in survival (Fig. 3)—as would occur if squirrels were reducing resources the mice required, but this model had little support. The vast majority (>99%) of the evidentiary weight fell behind models with site-specific estimates of survival. Upon closer examination of the effect of squirrels on mouse survival in five sites with large numbers of captures this effect appears to have been due to differences among sites rather than strong effects within sites (except for site 22). Similarly, there was strong support for models in which the capture probability of squirrels increased with chipmunk abundance and decreased with mouse abundance (>99% of the evidentiary weight), which could be evidence of competitors changing space use or activity levels of squirrels. However, when these models were fit individually to three sites with large numbers of captures the effects largely disappeared. Moreover, these models had virtually no support in the other set of sites, which calls into question the generality of these effects.

Lastly, there was virtually no evidence that the mass of the three focal species was negatively affected by the density of the other species as one might expect if they were competing for common food resources. Thus, even at this level, there is little evidence for strong interactions between squirrels, mice, and chipmunks.

It is possible that competitive interactions are only apparent when resources are scarce relative to consumer densities, either in space or time. After a mast year, for instance, population densities of mice and sciurid rodents tend to be very high [23], [24], perhaps beyond what the habitat can currently sustain, and so competition might be particularly intense in years following large masts. However, our long-term studies of acorn production in Dutchess County, NY indicated that acorn density was near the long-term average (9 acorns per m^2^; RSO unpublished data) in 2008, the year before our study began. Most of the trees in these fragments disperse seed in the fall, so we would expect their seeds to have been depleted before our study began, but these three species have rather broad diets and were presumably able to find adequate food resources. Alternatively, poor quality habitats might precipitate stronger competitive interactions. We were able to study 19 fragments, however, with little evidence of competition, which suggests that strong interactions are rare, at least in the summer.

We were only able to observe and manipulate these species from late spring through early fall. It is possible that these species do, in fact, interact and compete during the late fall, winter, and early spring when resources might sometimes be more limiting. Some food-addition studies with small rodents have generated only weak to no population increases when supplemental food is provided in summer [52], which suggests that summer food is not strongly limiting. However, other studies [53], [54] have shown that supplemental foods provided during the breeding season (spring and summer) can strongly increase *Peromyscus* densities. Dietary overlap between mice, chipmunks, and squirrels might also be lower during summer than during other seasons. Nevertheless, competitive interactions between rodents need not be caused solely by limited food resources. For instance, *Microtus* voles and *Peromyscus* mice show inversely related abundances and distributions in old fields despite little dietary overlap [55], [56]. And because the three rodents in our study share predators and parasites [57], the potential exists for apparent competition [58] to cause their densities to be inversely related. But otherwise, contrary to our expectations, we can conclude that white-footed mice, eastern chipmunks, and gray squirrels do not commonly or strongly affect each other's activity, apparent survival, reproductive effort, or density.

With only weak or nonexistent competition among these three common rodent species, we expect both habitat occupancy patterns and population dynamics of each species to be largely independent of the other two species. Our findings therefore support the conclusions of Moore and Swihart [25], whose habitat occupancy models suggested idiosyncratic responses by these three species to landscape patterns. We suggest that multifactorial models of population regulation of these three rodent species can be simplified to de-emphasize interspecific competition and focus more strongly on food, predators, and parasites.

## Supporting Information

Protocol S1 **Detailed information on the overall trapping schedule and exceptions to that schedule.**
(DOCX)

Table S1 How sites were assigned to removal and recipient/addition treatments, or to the control treatment.
(DOCX)

Table S2 The AICc-related metrics of fit of the Huggins robust design models used to examine the effects of chipmunk and squirrel abundance on the apparent survival and capture probability of mice.
(DOCX)

Table S3 The AICc-related metrics of fit of the Huggins robust design models used to examine the effects of mouse and chipmunk abundance on the apparent survival and capture probability of squirrels.
(DOCX)

Table S4 The AICc-related metrics of fit of the Huggins robust design models used to examine the effects of mouse and squirrel abundance on the apparent survival and capture probability of chipmunks.
(DOCX)
