# Supplementary material for: An Experimental Test of Competition among Mice, Chipmunks, and Squirrels in Deciduous Forest Fragments
Source: PLoS One. 2013 Jun 18;8(6):e66798. doi: 10.1371/journal.pone.0066798 (PMC3688938; doi:10.1371/journal.pone.0066798)
Supplement: Protocol S1 — Detailed information on the overall trapping schedule and exceptions to that schedule. (DOCX) [file pone.0066798.s001.docx]

This study involved trapping 19 forest fragments that were randomly assigned to 1 of 5 treatments, either mouse removal, mouse addition, squirrel removal, squirrel additions, or unmanipulated controls (Table S1). Generally, removal sites were trapped on Tuesdays and Wednesdays, dubbed “A” sites, and addition and control sites were trapped Thursdays and Friday, the “B” sites. The exceptions to this schedule are as follows:

• On June 19th site 67 (squirrel addition) was not trapped due to flooding.

• Trapping on 2609 (mouse addition) did not start until June 25th because we did not have permission until then.

• Trapping on addition and control sites did not take place on July 2nd and 3rd because of the holiday.

• Trapping on addition and control sites did not take place on July 9th and 10th for logistical reasons.

• Site 2709 (control) was originally trapped on Thursdays and Fridays, but starting on July 14th it was trapped on Tuesdays and Wednesdays. In order to simplify the mark-recapture analyses, this site was treated as if it was always trapped early in the week.

• On August 3rd (Tuesday) the crew accidentally set 2609 (mouse addition) instead of 2709 (control). Animals in these traps were release on August 4th (Wednesday) and were not considered in the analyses. We trapped 2709 on August 5th and 6th (Wednesday and Thursday) and 2609 on August 6th and 7th (Thursday and Friday). To simplify the mark-recapture analyses, these sites were treated as if they were trapped on the days they should have been.

• Each site was only trapped 2 days out of the last 2 weeks of trapping due to dwindling crew member numbers. Animals were not removed from removal sites on the first day of this last trapping session so that we could estimate the recapture probability within a trapping session and thus estimate the final abundances of mice and squirrels on removal sites. On the second day of this trapping session, eligible animals were removed to Freedom Forest. The schedule for the last 2 trapping weeks was as follows:

• Sites 32, 36, 40, 1009, 1109, and 3709 were trapped on September 22nd and 23rd.

• Sites 2, 5, 51, 209, 2609, and 2709 were trapped on September 24th and 25th.

• Sites 37, 39, and 67 were trapped on September 29th and 30th.

• Sites 19, 22, 909, and 1309 were trapped on October 1st and 2nd.

Each removal site was paired with an addition (=recipient) site as shown in Table S1. Removed animals would be transferred to addition site the same day as they were captured.
