# Supplementary material for: An Experimental Test of Competition among Mice, Chipmunks, and Squirrels in Deciduous Forest Fragments
Source: PLoS One. 2013 Jun 18;8(6):e66798. doi: 10.1371/journal.pone.0066798 (PMC3688938; doi:10.1371/journal.pone.0066798)
Supplement: Table S1 — How sites were assigned to removal and recipient/addition treatments, or to the control treatment. (DOCX) [file pone.0066798.s002.docx]

Table S1. The treatments to which 19 forest fragments in Dutchess County, New York were assigned. Note that the numbers identify the sites, not the number of animals added or removed. Animals removed from removal sites were added to their paired addition sites in the same row.

|  | Removal | Addition |
| --- | --- | --- |
| Mouse | 32 | 19 |
| Mouse | 40 | 39 |
| Mouse | 51 | 2609 |
| Mouse | 1109 | 22 |
| Squirrel | 36 | 37 |
| Squirrel | 209 | 2 |
| Squirrel | 1009 | 909 |
| Squirrel | 3709 | 67 |
|  |  |  |
| control | 5 | |
| control | 1309 | |
| control | 2709 | |
