# Supplementary material for: An Experimental Test of Competition among Mice, Chipmunks, and Squirrels in Deciduous Forest Fragments
Source: PLoS One. 2013 Jun 18;8(6):e66798. doi: 10.1371/journal.pone.0066798 (PMC3688938; doi:10.1371/journal.pone.0066798)
Supplement: Table S2 — The AICc-related metrics of fit of the Huggins robust design models used to examine the effects of chipmunk and squirrel abundance on the apparent survival and capture probability of mice. (DOCX) [file pone.0066798.s003.docx]

**Table S2**: Support for Huggins robust design mark-recapture models fit to mice data sets. Abbreviations are: k, the number of parameters; AICc, Akaike’s information criterion corrected for sample size; ∆*_i_*, difference in AICc; w_i_, AIC weight; sqrl, squirrel abundance; chip, chipmunk abundance; and trt, treatment. Parameters that are constant are represented by (.). Note that all models allowed for temporary Markovian immigration and emigration (*γ*” ≠ *γ*’).

| Models fit to removal sites | k | AICc | ∆*_i_* | w_i_ | Models fit to control and addition sites | k | AICc | ∆*_i_* | w_i_ |
| --- | --- | --- | --- | --- | --- | --- | --- | --- | --- |
| S(site) p(session+c) | 31 | 5721.7 | 0 | 0.999 | S(site) p(site+c) | 23 | 11176.1 | 0 | 0.999 |
| S(site) p(site+c) | 21 | 5736.1 | 14.5 | 0.001 | S(sqrl+chip) p(site+c) | 16 | 11190.7 | 14.6 | 0.001 |
| S(sqrl+chip) p(session+c) | 25 | 5746 | 24.4 | 0 | S(*ln*(sqrl)+*ln*(chip)) p(site+c) | 16 | 11193.6 | 17.5 | 0 |
| S(*ln*(sqrl)+*ln*(chip)) p(session+c) | 25 | 5747.7 | 26.1 | 0 | S(trt) p(site+c) | 16 | 11211.8 | 35.8 | 0 |
| S(.) p(session+c) | 23 | 5751.4 | 29.8 | 0 | S(sqrl+chip) p(session+c) | 22 | 11225.4 | 49.3 | 0 |
| S(trt) p(session+c) | 25 | 5751.5 | 29.9 | 0 | S(site) p(session+c) | 29 | 11225.5 | 49.4 | 0 |
| S(sqrl+chip) p(site+c) | 15 | 5756.1 | 34.4 | 0 | S(*ln*(sqrl)+*ln*(chip)) p(session+c) | 22 | 11226.2 | 50.1 | 0 |
| S(*ln*(sqrl)+*ln*(chip)) p(site+c) | 15 | 5757.3 | 35.6 | 0 | S(.) p(site+c) | 14 | 11227.3 | 51.2 | 0 |
| S(.) p(site+c) | 13 | 5757.9 | 36.2 | 0 | S(trt) p(session+c) | 22 | 11256.4 | 80.4 | 0 |
| S(trt) p(site+c) | 15 | 5759.5 | 37.8 | 0 | S(.) p(session+c) | 20 | 11269.8 | 93.7 | 0 |
| S(site) p(trt+c) | 15 | 5783 | 61.3 | 0 | S(site) p(trt+c) | 16 | 11321.1 | 145 | 0 |
| S(site) p(sqrl+chip+c) | 15 | 5793.1 | 71.4 | 0 | S(sqrl+chip) p(trt+c) | 9 | 11334.6 | 158.5 | 0 |
| S(site) p(*ln*(sqrl)+*ln*(chip)+c) | 15 | 5795.5 | 73.8 | 0 | S(site) p(sqrl+chip+c) | 16 | 11338.7 | 162.6 | 0 |
| S(site) p(.+c) | 13 | 5802 | 80.3 | 0 | S(*ln*(sqrl)+*ln*(chip)) p(trt+c) | 9 | 11338.8 | 162.7 | 0 |
| S(sqrl+chip) p(trt+c) | 9 | 5806.9 | 85.3 | 0 | S(site) p(.+c) | 14 | 11341.4 | 165.3 | 0 |
| S(*ln*(sqrl)+*ln*(chip)) p(trt+c) | 9 | 5808 | 86.3 | 0 | S(site) p(*ln*(sqrl)+*ln*(chip)+c) | 16 | 11344 | 167.9 | 0 |
| S(.) p(trt+c) | 7 | 5809.6 | 87.9 | 0 | S(sqrl+chip) p(sqrl+chip+c) | 9 | 11349.5 | 173.4 | 0 |
| S(trt) p(trt+c) | 9 | 5810.8 | 89.1 | 0 | S(trt) p(trt+c) | 9 | 11351.4 | 175.3 | 0 |
| S(sqrl+chip) p(sqrl+chip+c) | 9 | 5818 | 96.3 | 0 | S(sqrl+chip) p(.+c) | 7 | 11352 | 175.9 | 0 |
| S(.) p(sqrl+chip+c) | 7 | 5820.8 | 99.1 | 0 | S(*ln*(sqrl)+*ln*(chip)) p(.+c) | 7 | 11355.6 | 179.5 | 0 |
| S(*ln*(sqrl)+*ln*(chip)) p(*ln*(sqrl)+*ln*(chip)+c) | 9 | 5821 | 99.3 | 0 | S(*ln*(sqrl)+*ln*(chip)) p(*ln*(sqrl)+*ln*(chip)+c) | 9 | 11358.3 | 182.2 | 0 |
| S(trt) p(sqrl+chip+c) | 9 | 5821.6 | 99.9 | 0 | S(.) p(trt+c) | 7 | 11368.6 | 192.5 | 0 |
| S(.) p(*ln*(sqrl)+*ln*(chip)+c) | 7 | 5822.5 | 100.8 | 0 | S(trt) p(sqrl+chip+c) | 9 | 11370.6 | 194.5 | 0 |
| S(trt) p(*ln*(sqrl)+*ln*(chip)+c) | 9 | 5823.4 | 101.7 | 0 | S(trt) p(.+c) | 7 | 11372.2 | 196.1 | 0 |
| S(sqrl+chip) p(.+c) | 7 | 5827.1 | 105.5 | 0 | S(trt) p(*ln*(sqrl)+*ln*(chip)+c) | 9 | 11375.3 | 199.2 | 0 |
| S(*ln*(sqrl)+*ln*(chip)) p(.+c) | 7 | 5828.3 | 106.6 | 0 | S(.) p(sqrl+chip+c) | 7 | 11385.6 | 209.5 | 0 |
| S(.) p(.+c) | 5 | 5830 | 108.3 | 0 | S(.) p(.+c) | 5 | 11387.2 | 211.2 | 0 |
| S(trt) p(.+c) | 7 | 5830.2 | 108.6 | 0 | S(.) p(*ln*(sqrl)+*ln*(chip)+c) | 7 | 11390.3 | 214.2 | 0 |
