# Supplementary material for: An Experimental Test of Competition among Mice, Chipmunks, and Squirrels in Deciduous Forest Fragments
Source: PLoS One. 2013 Jun 18;8(6):e66798. doi: 10.1371/journal.pone.0066798 (PMC3688938; doi:10.1371/journal.pone.0066798)
Supplement: Table S3 — The AICc-related metrics of fit of the Huggins robust design models used to examine the effects of mouse and chipmunk abundance on the apparent survival and capture probability of squirrels. (DOCX) [file pone.0066798.s004.docx]

**Table S3**: Support for Huggins robust design mark-recapture models fit to squirrel data sets. Abbreviations are: k the number of parameters; AICc Akaike’s information criterion corrected for sample size; ∆*_i_*, difference in AICc; and w_i_, AIC weight. Parameters that are constant are represented by (.). Note that all models allowed for temporary Markovian immigration and emigration (*γ*” ≠ *γ*’).

| Models fit to removal sites | k | AICc | ∆*_i_* | w*_i_* |  | Models fit to control & addition sites | k | AICc | ∆*_i_* | w*_i_* |
| --- | --- | --- | --- | --- | --- | --- | --- | --- | --- | --- |
| S(.) p(*ln*(mice)+*ln*(chip)+c) | 7 | 1374.9 | 0 | 0.792 |  | S(.) p(site+c) | 14 | 977.2 | 0 | 0.687 |
| S(*ln*(mice)+*ln*(chip)) p(*ln*(mice)+*ln*(chip)+c) | 9 | 1379.0 | 4.1 | 0.1 |  | S(*ln*(mice)+*ln*(chip)) p(site+c) | 16 | 981.2 | 4 | 0.094 |
| S(trt) p(*ln*(mice)+*ln*(chip)+c) | 9 | 1379.1 | 4.2 | 0.096 |  | S(mice+chip) p(site+c) | 16 | 981.7 | 4.5 | 0.074 |
| S(.) p(site+c) | 13 | 1384.3 | 9.4 | 0.007 |  | S(trt) p(site+c) | 16 | 981.8 | 4.5 | 0.071 |
| S(site) p(*ln*(mice)+*ln*(chip)+c) | 15 | 1387.5 | 12.6 | 0.001 |  | S(.) p(trt+c) | 7 | 984.0 | 6.8 | 0.023 |
| S(mice+chip) p(site+c) | 15 | 1388.5 | 13.6 | 0.001 |  | S(.) p(.+c) | 5 | 985.8 | 8.6 | 0.009 |
| S(*ln*(mice)+*ln*(chip)) p(site+c) | 15 | 1388.6 | 13.7 | 0.001 |  | S(*ln*(mice)+*ln*(chip)) p(trt+c) | 9 | 986.0 | 8.8 | 0.008 |
| S(trt) p(site+c) | 15 | 1388.6 | 13.7 | 0.001 |  | S(*ln*(mice)+*ln*(chip)) p(.+c) | 7 | 986.3 | 9.1 | 0.007 |
| S(.) p(mice+chip+c) | 7 | 1390.9 | 16 | 0 |  | S(mice+chip) p(trt+c) | 9 | 986.6 | 9.4 | 0.006 |
| S(.) p(session+c) | 23 | 1394.2 | 19.3 | 0 |  | S(mice+chip) p(.+c) | 7 | 986.7 | 9.4 | 0.006 |
| S(mice+chip) p(mice+chip+c) | 9 | 1394.4 | 19.5 | 0 |  | S(trt) p(trt+c) | 9 | 988.3 | 11.1 | 0.003 |
| S(trt) p(mice+chip+c) | 9 | 1394.8 | 19.9 | 0 |  | S(trt) p(.+c) | 7 | 988.3 | 11.1 | 0.003 |
| S(.) p(trt+c) | 7 | 1395.8 | 20.9 | 0 |  | S(.) p(mice+chip+c) | 7 | 988.7 | 11.5 | 0.002 |
| S(mice+chip) p(session+c) | 25 | 1398.3 | 23.4 | 0 |  | S(.) p(*ln*(mice)+*ln*(chip)+c) | 7 | 989.9 | 12.7 | 0.001 |
| S(trt) p(session+c) | 25 | 1398.6 | 23.7 | 0 |  | S(mice+chip) p(mice+chip+c) | 9 | 990.1 | 12.8 | 0.001 |
| S(*ln*(mice)+*ln*(chip)) p(session+c) | 25 | 1398.6 | 23.7 | 0 |  | S(*ln*(mice)+*ln*(chip)) p(*ln*(mice)+*ln*(chip)+c) | 9 | 990.2 | 12.9 | 0.001 |
| S(site) p(site+c) | 21 | 1398.8 | 23.9 | 0 |  | S(trt) p(mice+chip+c) | 9 | 991.4 | 14.2 | 0.001 |
| S(mice+chip) p(trt+c) | 9 | 1399.5 | 24.6 | 0 |  | S(trt) p(*ln*(mice)+*ln*(chip)+c) | 9 | 992.2 | 14.9 | 0 |
| S(.) p(.+c) | 5 | 1399.9 | 25 | 0 |  | S(site) p(.+c) | 14 | 993.6 | 16.4 | 0 |
| S(trt) p(trt+c) | 9 | 1399.9 | 25 | 0 |  | S(site) p(site+c) | 23 | 995.0 | 17.7 | 0 |
| S(*ln*(mice)+*ln*(chip)) p(trt+c) | 9 | 1400.0 | 25.1 | 0 |  | S(site) p(trt+c) | 16 | 995.3 | 18 | 0 |
| S(mice+chip) p(.+c) | 7 | 1403.1 | 28.2 | 0 |  | S(site) p(*ln*(mice)+*ln*(chip)+c) | 16 | 996.9 | 19.7 | 0 |
| S(*ln*(mice)+*ln*(chip)) p(.+c) | 7 | 1403.6 | 28.7 | 0 |  | S(site) p(mice+chip+c) | 16 | 997.1 | 19.8 | 0 |
| S(trt) p(.+c) | 7 | 1403.8 | 28.9 | 0 |  | S(.) p(session+c) | 20 | 1005.2 | 28 | 0 |
| S(site) p(mice+chip+c) | 15 | 1403.9 | 29 | 0 |  | S(*ln*(mice)+*ln*(chip)) p(session+c) | 22 | 1007.3 | 30 | 0 |
| S(site) p(trt+c) | 15 | 1408.5 | 33.6 | 0 |  | S(mice+chip) p(session+c) | 22 | 1007.3 | 30.1 | 0 |
| S(site) p(session+c) | 31 | 1408.8 | 33.9 | 0 |  | S(trt) p(session+c) | 22 | 1008.4 | 31.2 | 0 |
| S(site) p(.+c) | 13 | 1412.2 | 37.3 | 0 |  | S(site) p(session+c) | 29 | 1017.5 | 40.2 | 0 |
