# Supplementary material for: An Experimental Test of Competition among Mice, Chipmunks, and Squirrels in Deciduous Forest Fragments
Source: PLoS One. 2013 Jun 18;8(6):e66798. doi: 10.1371/journal.pone.0066798 (PMC3688938; doi:10.1371/journal.pone.0066798)
Supplement: Table S4 — The AICc-related metrics of fit of the Huggins robust design models used to examine the effects of mouse and squirrel abundance on the apparent survival and capture probability of chipmunks. (DOCX) [file pone.0066798.s005.docx]

**Table S4**: Support for Huggins robust design mark-recapture models fit to chipmunk data sets. Abbreviations are: k the number of parameters; AICc Akaike’s information criterion corrected for sample size; ∆*_i_*, difference in AICc; and w_i_, AIC weight. Parameters that are constant are represented by (.). Note that all models allowed for temporary Markovian immigration and emigration (*γ*” ≠ *γ*’).

| Models fit to removal sites | k | AICc | ∆*_i_* | w_i_ | Models fit to control & addition sites | k | AICc | ∆_i_ | w_i_ |
| --- | --- | --- | --- | --- | --- | --- | --- | --- | --- |
| S(.) p(site+c) | 13 | 2693.6 | 0 | 0.69 | S(site) p(site+c) | 21 | 2059.1 | 0 | 0.377 |
| S(*ln*(mice)+*ln*(sqrl)) p(site+c) | 15 | 2697.2 | 3.6 | 0.113 | S(.) p(site+c) | 13 | 2059.3 | 0.2 | 0.341 |
| S(trt) p(site+c) | 15 | 2697.4 | 3.8 | 0.105 | S(*ln*(mice)+*ln*(sqrl)) p(site+c) | 15 | 2061.4 | 2.3 | 0.117 |
| S(mice+sqrl) p(site+c) | 15 | 2697.7 | 4.1 | 0.09 | S(mice+sqrl) p(site+c) | 15 | 2062.6 | 3.5 | 0.065 |
| S(site) p(site+c) | 21 | 2706.1 | 12.5 | 0.001 | S(trt) p(site+c) | 15 | 2062.6 | 3.6 | 0.063 |
| S(.) p(trt+c) | 7 | 2749.9 | 56.3 | 0 | S(.) p(session+c) | 20 | 2065.3 | 6.2 | 0.017 |
| S(*ln*(mice)+*ln*(sqrl)) p(trt+c) | 9 | 2752.3 | 58.7 | 0 | S(site) p(session+c) | 28 | 2066.7 | 7.6 | 0.008 |
| S(.) p(mice+sqrl+c) | 7 | 2752.8 | 59.2 | 0 | S(*ln*(mice)+*ln*(sqrl)) p(session+c) | 22 | 2067.8 | 8.7 | 0.005 |
| S(trt) p(mice+sqrl+c) | 9 | 2752.9 | 59.3 | 0 | S(trt) p(session+c) | 22 | 2067.9 | 8.8 | 0.005 |
| S(trt) p(trt+c) | 9 | 2753.6 | 60 | 0 | S(mice+sqrl) p(session+c) | 22 | 2069.2 | 10.1 | 0.002 |
| S(mice+sqrl) p(trt+c) | 9 | 2753.9 | 60.3 | 0 | S(.) p(trt+c) | 7 | 2071.7 | 12.6 | 0.001 |
| S(.) p(.+c) | 5 | 2755.6 | 62 | 0 | S(site) p(trt+c) | 15 | 2073.2 | 14.2 | 0 |
| S(site) p(trt+c) | 15 | 2755.6 | 62 | 0 | S(*ln*(mice)+*ln*(sqrl)) p(trt+c) | 9 | 2074.3 | 15.2 | 0 |
| S(site) p(mice+sqrl+c) | 15 | 2756 | 62.4 | 0 | S(trt) p(trt+c) | 9 | 2074.6 | 15.6 | 0 |
| S(trt) p(.+c) | 7 | 2756.4 | 62.8 | 0 | S(mice+sqrl) p(trt+c) | 9 | 2075.4 | 16.4 | 0 |
| S(mice+sqrl) p(mice+sqrl+c) | 9 | 2756.7 | 63.1 | 0 | S(.) p(mice+sqrl+c) | 7 | 2091.3 | 32.3 | 0 |
| S(*ln*(mice)+*ln*(sqrl)) p(.+c) | 7 | 2758.2 | 64.6 | 0 | S(site) p(mice+sqrl+c) | 15 | 2091.4 | 32.4 | 0 |
| S(site) p(.+c) | 13 | 2758.3 | 64.7 | 0 | S(trt) p(mice+sqrl+c) | 9 | 2094.4 | 35.3 | 0 |
| S(.) p(*ln*(mice)+*ln*(sqrl)+c) | 7 | 2759.3 | 65.7 | 0 | S(mice+sqrl) p(mice+sqrl+c) | 9 | 2094.7 | 35.7 | 0 |
| S(mice+sqrl) p(.+c) | 7 | 2759.7 | 66.1 | 0 | S(.) p(.+c) | 5 | 2095.4 | 36.3 | 0 |
| S(trt) p(*ln*(mice)+*ln*(sqrl)+c) | 9 | 2760 | 66.4 | 0 | S(site) p(.+c) | 13 | 2096.4 | 37.3 | 0 |
| S(*ln*(mice)+*ln*(sqrl)) p(*ln*(mice)+*ln*(sqrl)+c) | 9 | 2761.9 | 68.3 | 0 | S(.) p(*ln*(mice)+*ln*(sqrl)+c) | 7 | 2096.6 | 37.6 | 0 |
| S(site) p(*ln*(mice)+*ln*(sqrl)+c) | 15 | 2761.9 | 68.3 | 0 | S(site) p(*ln*(mice)+*ln*(sqrl)+c) | 15 | 2097 | 37.9 | 0 |
| S(.) p(session+c) | 23 | 2763.1 | 69.5 | 0 | S(*ln*(mice)+*ln*(sqrl)) p(.+c) | 7 | 2097.2 | 38.2 | 0 |
| S(trt) p(session+c) | 25 | 2764.5 | 70.9 | 0 | S(trt) p(.+c) | 7 | 2097.8 | 38.7 | 0 |
| S(*ln*(mice)+*ln*(sqrl)) p(session+c) | 25 | 2765.2 | 71.6 | 0 | S(*ln*(mice)+*ln*(sql)) p(*ln*(mice)+*ln*(sqrl)+c) | 9 | 2098.2 | 39.1 | 0 |
| S(site) p(session+c) | 31 | 2767.2 | 73.6 | 0 | S(mice+sqrl) p(.+c) | 7 | 2098.6 | 39.5 | 0 |
| S(mice+sqrl) p(session+c) | 25 | 2767.3 | 73.7 | 0 | S(trt) p(*ln*(mice)+*ln*(sqrl)+c) | 9 | 2099.3 | 40.2 | 0 |
